# Supplementary material for: Bayesian refinement of protein structures and ensembles against SAXS data using molecular dynamics
Source: PLoS Comput Biol. 2017 Oct 18;13(10):e1005800. doi: 10.1371/journal.pcbi.1005800 (PMC5662244; doi:10.1371/journal.pcbi.1005800)
Supplement: S1 Archive — (BZ2) [file pcbi.1005800.s011.tar.bz2 › gromacs_4.62_bayesian_saxs_refi/share/html/online/g_rms.html]

g\_rms


| g\_rms Main Table of Contents |

**VERSION 4.6  
Sat 19 Jan 2013**

---

### Description

g\_rms compares two structures by computing the root mean square
deviation (RMSD), the size-independent ρ similarity parameter
(rho) or the scaled ρ (rhosc),
see Maiorov & Crippen, Proteins **22**, 273 (1995).
This is selected by -what.

Each structure from a trajectory (-f) is compared to a
reference structure. The reference structure
is taken from the structure file (-s).

With option -mir also a comparison with the mirror image of
the reference structure is calculated.
This is useful as a reference for 'significant' values, see
Maiorov & Crippen, Proteins **22**, 273 (1995).

Option -prev produces the comparison with a previous frame
the specified number of frames ago.

Option -m produces a matrix in .xpm format of
comparison values of each structure in the trajectory with respect to
each other structure. This file can be visualized with for instance
xv and can be converted to postscript with xpm2ps.

Option -fit controls the least-squares fitting of
the structures on top of each other: complete fit (rotation and
translation), translation only, or no fitting at all.

Option -mw controls whether mass weighting is done or not.
If you select the option (default) and
supply a valid .tpr file masses will be taken from there,
otherwise the masses will be deduced from the atommass.dat file in
GMXLIB. This is fine for proteins, but not
necessarily for other molecules. A default mass of 12.011 amu (carbon)
is assigned to unknown atoms. You can check whether this happend by
turning on the -debug flag and inspecting the log file.

With -f2, the 'other structures' are taken from a second
trajectory, this generates a comparison matrix of one trajectory
versus the other.

Option -bin does a binary dump of the comparison matrix.

Option -bm produces a matrix of average bond angle deviations
analogously to the -m option. Only bonds between atoms in the
comparison group are considered.

### Files

| option | filename | type | description |
| --- | --- | --- | --- |
| **-s** | topol.tpr | Input | Structure+mass(db): tpr tpb tpa gro g96 pdb |
| **-f** | traj.xtc | Input | Trajectory: xtc trr trj gro g96 pdb cpt |
| **-f2** | traj.xtc | Input, Opt. | Trajectory: xtc trr trj gro g96 pdb cpt |
| **-n** | index.ndx | Input, Opt. | Index file |
| **-o** | rmsd.xvg | Output | xvgr/xmgr file |
| **-mir** | rmsdmir.xvg | Output, Opt. | xvgr/xmgr file |
| **-a** | avgrp.xvg | Output, Opt. | xvgr/xmgr file |
| **-dist** | rmsd-dist.xvg | Output, Opt. | xvgr/xmgr file |
| **-m** | rmsd.xpm | Output, Opt. | X PixMap compatible matrix file |
| **-bin** | rmsd.dat | Output, Opt. | Generic data file |
| **-bm** | bond.xpm | Output, Opt. | X PixMap compatible matrix file |

### Other options

| option | type | default | description |
| --- | --- | --- | --- |
| **-[no]h** | bool | no | Print help info and quit || **-[no]version** | bool | no | Print version info and quit || **-nice** | int | 19 | Set the nicelevel || **-b** | time | 0 | First frame (ps) to read from trajectory || **-e** | time | 0 | Last frame (ps) to read from trajectory || **-dt** | time | 0 | Only use frame when t MOD dt = first time (ps) || **-tu** | enum | ps | Time unit: fs, ps, ns, us, ms or s || **-[no]w** | bool | no | View output .xvg, .xpm, .eps and .pdb files || **-xvg** | enum | xmgrace | xvg plot formatting: xmgrace, xmgr or none || **-what** | enum | rmsd | Structural difference measure: rmsd, rho or rhosc || **-[no]pbc** | bool | yes | PBC check || **-fit** | enum | rot+trans | Fit to reference structure: rot+trans, translation or none || **-prev** | int | 0 | Compare with previous frame || **-[no]split** | bool | no | Split graph where time is zero || **-skip** | int | 1 | Only write every nr-th frame to matrix || **-skip2** | int | 1 | Only write every nr-th frame to matrix || **-max** | real | -1 | Maximum level in comparison matrix || **-min** | real | -1 | Minimum level in comparison matrix || **-bmax** | real | -1 | Maximum level in bond angle matrix || **-bmin** | real | -1 | Minimum level in bond angle matrix || **-[no]mw** | bool | yes | Use mass weighting for superposition || **-nlevels** | int | 80 | Number of levels in the matrices || **-ng** | int | 1 | Number of groups to compute RMS between |

---

http://www.gromacs.org  
gromacs@gromacs.org
